# A Comparison of In-House Real-Time LAMP Assays with a Commercial Assay for the Detection of Pathogenic Bacteria

**DOI:** 10.3390/molecules20069487

**Published:** 2015-05-25

**Authors:** Deguo Wang, Yongzhen Wang, Fugang Xiao, Weiyun Guo, Yongqing Zhang, Aiping Wang, Yanhong Liu

**Affiliations:** 1 Henan Postdoctoral Research Base, Food and Bioengineering College, Xuchang University, Xuchang 461000, Henan, China; E-Mails: wangdg666@126.com (D.W.); xfug@163.com (F.X.); gwy2002@126.com (W.G.); zyq336@163.com (Y.Z.); 2 Public Lab Center, Xuchang University, Xuchang 461000, Henan, China; E-Mail: wangyzhxuch@sina.com; 3 Life Science College, Zhengzhou University, Zhengzhou 450000, Henan, China; E-Mail: pingaw@126.com; 4 Molecular Characterization of Foodborne Pathogens Research Unit, Eastern Regional Research Center, Agricultural Research Service, United States Department of Agriculture, Wyndmoor, PA 19038, USA

**Keywords:** real-time loop-mediated isothermal amplification (LAMP), specificity, sensitivity, pathogenic bacteria

## Abstract

Molecular detection of bacterial pathogens based on LAMP methods is a faster and simpler approach than conventional culture methods. Although different LAMP-based methods for pathogenic bacterial detection are available, a systematic comparison of these different LAMP assays has not been performed. In this paper, we compared 12 in-house real-time LAMP assays with a commercialized kit (Isothermal Master Mix) for the detection of *Listeria monocytogenes*, *Salmonella* spp, *Staphylococcus aureus*, *Escherichia coli* O157, *E. coli* O26, *E. coli* O45, *E. coli* O103, *E. coli* O111, *E. coli* O121, *E. coli* O145 and *Streptococcus agalactiae*. False-positive results were observed in all 12 in-house real-time LAMP assays, while all the negative controls of Isothermal Master Mix remained negative after amplification. The detection limit of Isothermal Master Mix for *Listeria monocytogenes*, *Salmonella* spp, *Staphylococcus aureus*, *Escherichia coli* O157, *E. coli* O26, *E. coli* O45, *E. coli* O103, *E. coli* O111, *E. coli* O121 and *Streptococcus agalactiae* was 1 pg, whereas the sensitivity of the commercialized kit for *E. coli* O145 was 100 pg. In conclusion, the 12 in-house real-time LAMP assays were impractical to use, while the commercialized kit Isothermal Master Mix was useful for the detection of most bacterial pathogens.

## 1. Introduction

Loop-mediated isothermal amplification (LAMP), developed by Notomi *et al.* in 2000 [1], can specifically, sensitively and rapidly amplify nucleic acids utilizing a DNA polymerase enzyme with high strand displacement activity and two pairs of primers recognizing six independent sequences of a target gene under isothermal conditions. Moreover, Nagamine *et al.* in 2002 has advanced the method by incorporating forward loop primers that accelerate the LAMP reaction [2]. Due to the cost effectiveness and sensitivity of LAMP, this method has been widely applied to basic medical research in and environmental testing, as well as point-of-care testing and diagnosis of infectious diseases in clinical settings [3]. In addition, LAMP has also been applied for pathogen detection, and has successfully been used to detect *Listeria monocytogenes* [4], *Salmonella*, spp [5], *Staphylococcus aureus* [6], *Escherichia coli* O157 [7,8], *E. coli* O26 [7], *E. coli* O45 [7], *E. coli* O103 [7], *E. coli* O111 [7], *E. coli* O121 [7], *Streptococcus agalactiae* [9], *Actinobacillus actinomycetemcomitans* [10], *Mycobacterium tuberculosis* [11], and *Streptococcus pneumonia* [12]. However, a systematic evaluation of these different LAMP assays has not been performed.

The objective of the present study was to compare 12 in-house real-time LAMP assays for the detection of *Listeria monocytogenes*, *Salmonella*, *Staphylococcus aureus*, *Escherichia coli* O157, *E. coli* O26, *E. coli* O45, *E. coli* O103, *E. coli* O111, *E. coli* O121, *E. coli* O145 and *Streptococcus agalactiae* performed using previously described protocols [4,5,6,7,8,9] with a commercial kit (Isothermal Master Mix, OptiGene, Horsham, UK).

## 2. Results and Discussion

### 2.1. Non-Specific Amplification

After the negative controls without genomic DNA were kept at the described temperature for 50 min, false-positive results were observed among all 12 in-house real-time LAMP assays for detection of *L. monocytogenes*, *Salmonella*, *Staphylococcus aureus*, *E. coli* O157, *E. coli* O26, *E. coli* O45, *E. coli* O103, *E. coli* O111, *E. coli* O121 and *Streptococcus agalactiae*, while all negative controls of the commercial kit Isothermal Master Mix remained negative after amplification at 65 °C for 50 min, as indicated in Table 1. The cross pollution caused by DNA templates as well as amplified products of the LAMP reactions had been excluded, therefore, the non-specific amplification of 12 in-house real-time LAMP assays could be caused by primer dimers, in contrast, the commercial assay can be free of such non-specific amplification.

**Table 1:** Results of non-specific amplification determination from in-house real-time LAMP assay and the isothermal master mix kit.

Bacterial Strains (Gene)	Assay	No. LAMP-Positive/No. of Total Experiments Carried Out
*Listeria monocytogenes* (*hly*A)	In-house Real-time LAMP Assay	3/3
	Isothermal Master Mix	0/3
*Salmonella* spp (*inv*A)	In-house Real-time LAMP Assay	2/3
	Isothermal Master Mix	0/3
*Staphylococcus aureus* (*nuc*)	In-house Real-time LAMP Assay	3/3
	Isothermal Master Mix	0/3
*Escherichia coli* O157 (*rfb*E)	In-house Real-time LAMP Assay	1/3
	Isothermal Master Mix	0/3
*Escherichia coli* O26 (*wzy*)	In-house Real-time LAMP Assay	3/3
	Isothermal Master Mix	0/3
*Escherichia coli* O45 (*wzy*)	In-house Real-time LAMP Assay	3/3
	Isothermal Master Mix	0/3
*Escherichia coli* O103 (*wzx*)	In-house Real-time LAMP Assay	1/3
	Isothermal Master Mix	0/3
*Escherichia coli* O111 (*wzy*)	In-house Real-time LAMP Assay	2/3
	Isothermal Master Mix	0/3
*Escherichia coli* O121 (*wzy*)	In-house Real-time LAMP Assay	2/3
	Isothermal Master Mix	0/3
*Escherichia coli* O145 (*wzx*)	In-house Real-time LAMP Assay	3/3
	Isothermal Master Mix	0/3
*Escherichia coli* O157 (wzy)	In-house Real-time LAMP Assay	3/3
	Isothermal Master Mix	0/3
*Streptococcus agalactiae* (*sob*A)	In-house Real-time LAMP Assay	2/3
	Isothermal Master Mix	0/3

### 2.2. Sensitivity of the Commercial Assay

Because of the serious non-specific amplification, determining the detection limits of the 12 in-house real-time LAMP assays was of no practical significance, and only the sensitivity of the commercial Isothermal Master Mix kit for pathogenic bacteria detection was determined.

As Table 2 indicates, the detection limit of Isothermal Master Mix for *Listeria monocytogenes*, *Salmonella* spp, *Staphylococcus aureus*, *Escherichia coli* O157, *E. coli* O26, *E. coli* O45, *E. coli* O103, *E. coli* O111, *E. coli* O121 and *Streptococcus agalactiae* was 1 pg. However, all reactions with serial dilutions of *E. coli* O145 DNA template ranging from 0.1–100 pg were negative, and then we repeated the experiment, one of four reactions with 100 pg *E. coli* O145 DNA template was positive (data not shown), therefore, the LAMP assay with the commercial kit Isothermal Master may not suitable for detection of some pathogenic bacteria because of the low sensitivity.

**Table 2:** Sensitivity of real-time LAMP assays with the Isothermal Master Mix Kit.

Bacterial Strains (Gene) ^a^	10 pg DNA Template	1 pg DNA Template	0.1 pg DNA Template	Negative Controls
*Listeria monocytogenes* ATCC19115 (*hly*A)	4/4	4/4	0/4	0/4
*Salmonella enterica* serotype Newport (*inv*A)	4/4	4/4	0/4	0/4
*Staphylococcus aureus* ATCC 25923 (*nuc*)	4/4	4/4	0/4	0/4
*Escherichia coli* O157:H7 933 (*rfb*E)	4/4	4/4	0/4	0/4
*Escherichia coli* O26:H11 (*wzy*)	4/4	3/4	1/4	0/4
*Escherichia coli* O45:H12 (*wzy*)	4/4	2/4	0/4	0/4
*Escherichia coli* O103:H2 (*wzx*)	4/4	4/4	0/4	0/4
*Escherichia coli* O111:H8 (*wzy*)	4/4	4/4	0/4	0/4
*Escherichia coli* O121:H19 (*wzy*)	4/4	2/4	0/4	0/4
*Escherichia coli* O145:H2 (*wzx*)	1/8	0/8	0/8	0/8
*Escherichia coli* O157:H7 933 (*wzy*)	4/4	4/4	0/4	0/4
*Streptococcus agalactiae* ATCC 27956 (*sob*A)	4/4	4/4	0/4	0/4

^a^ All these strains were obtained from the Food and Bioengineering College of Xuchang University, Henan, China.

## 3. Experimental Section

### 3.1. LAMP Primers

The described LAMP primers targeting specific genes of the *hly*A of *Listeria monocytogenes*, *inv*A of *Salmonella*, *nuc* of *Staphylococcus aureus*, *rfb*E of *Escherichia coli* O157, *wzy* of *E. coli* O26, *wzy* of *E. coli* O45, *wzx* of *E. coli* O103, *wzy* of *E. coli* O111, *wzy* of *E. coli* O121, *wzx* of *E. coli* O145, *wzy* of *rfb*E of *E. coli* O157 and *sobA* of *Streptococcus agalactiae* used in this study are shown in Table 3 [4,5,6,7,8,9].

**Table 3:** Primers of the 12 in-house real-time LAMP assays for bacterial pathogen detection.

Bacterial Strains	Gene	Primer	Sequence (5*'*-3*'*)	References
*Listeria monocytogenes*	*hly*A	LB	GCCAAGAAAAGGTTACAAAGATGG	[4]
		LF	TAGGACTTGCAGGCGGAGATG	
		B3	GCTTTTACGAGAGCACCTGG	
		F3	TTGCGCAACAAACTGAAGC	
		BIP	CCACGGAGATGCAGTGACAAATGTTTTGGATTTCTTCTTTTTCTCCACAAC	
		FIP	CGTGTTTCTTTTCGATTGGCGTCTTTTTTTCATCCATGGCACCACC	
*Salmonella spp*	*inv*A	LB	GGGCAATTCGTTATTGGCGATAG	[5]
		LF	GACGAAAGAGCGTGGTAATTAAC	
		B3	AACGATAAACTGGACCACGG	
		F3	GGCGATATTGGTGTTTATGGGG	
		BIP	CCGGTGAAATTATCGCCACACAAAACCCACCGCCAGG	
		FIP	GACGACTGGTACTGATCGATAGTTTTTCAACGTTTCCTGCGG	
*Staphylococcus aureus*	*nuc*	LB	CAAACCTAACAATACACATGAACA	[6]
		LF	ACGCTAAGCCACGTCCATAT	
		B3	CGTTGTCTTCGCTCCAAAT	
		F3	TGCAAAGAAAATTGAAGTCGA	
		BIP	TCAAGGCTTGGCTAAAGTTGCTTATTCGCTTGTGCTTCACTT	
		FIP	CGTTTACCATTTTTCCATCAGCATATTTGACAAAGGTCAAAGAACT	
*Escherichia coli* O157	*rfb*E	B3	GGTGCTTTTGATATTTTTCCG	[7]
		F3	AACAGTCTTGTACAAGTCCA	
		BIP	CTCTCTTTCCTCTGCGGTCC-GATGTTTTTCACACTTATTGGAT	
		FIP	TAAGGAATCACCTTGCAGATAAACT-AGTACATTGGCATCGTGT	
*Escherichia coli* O26	*wzy*	LB	TACAATACAGTAAGTATACAGCATT	[8]
		LF	ACCAGCGATAACCAATCTC	
		B3	TCCTGATTTGAACAATGTCAAT	
		F3	GACTATGAAGCGTATGTTGAT	
		BIP	TTCCTTGGGACCACATTCCT-ACATGTAAAGCAGCAAACC	
		FIP	ACCGCCTAAATACTTAACACCATAA-TTAATGTCAATGAACTTTATGCC	
*Escherichia coli* O45	*wzy*	LB	TTATTACTCCTGGCAGTATTAATCG	[8]
		B3	TTTAGTCGCTCGCCAAGA	
		F3	AATGTCCCCAGGGTTTGT	
		BIP	AGCGGGCTAATATTAGTAGTCACTC-GTATGCTTCAATTTGGCTGT	
		FIP	ACTCTGGGTTTGATTTTTTCACTTC-ATAATTTCATCCAGACGAACG	
*Escherichia coli* O103	*wzx*	LB	CCTTTATAAATGGATTCATTTCATC	[8]
		LF	AATTGCAACAACTTTTGAAATAA	
		B3	TCACCTTGATTTTCTGCTGA	
		F3	ACTCAGTGGTGTAGTAACATG	
		BIP	TTGGGACAATTGCAAAATTTTGTGG-ATCTATTAACTCCTTGTGAAACTTG	
		FIP	ATTTGCTATTCCAATTGGACCAGTA-CTTTAGACTAATTTGTGGCCTTC	
*Escherichia coli* O111	*wzy*	LB	CTTAAATAACGGCGGACAAT	[8]
		B3	TCATGAGGGTCATTAGGAATT	
		F3	AAGGCGTAACTTTTTTTGAAC	
		BIP	TCCATGGTATGGGGACATTAAATTT-TGATGGAAGTCCATATAACGT	
		FIP	TCACCAAGCTGTGAAACCAAA-CTACAGCAAGTAATATTGAACGT	
*Escherichia coli* O121	*wzy*	LF	TAAAGCCATCCAACCACGC	[8]
		B3	ATAGGCTCCCAACCATCC	
		F3	GCTCAGCTTTTATCTTGTTCAA	
		BIP	TGTTGCTGGTTCCTTATTATGTAGT-AAAAGCAAGCCAAAACACTC	
		FIP	ACGCAAAAAGTATGGATTCATACCT-GATATAACAGAACCGACTTGG	
*Escherichia coli* O145	*wzx*	LF	TTCTTAAGTTCGGATACACTAGCA	[8]
		B3	GCATTGGTACAGACAGCTTTA	
		F3	TTTGTAAGACAAGGTGTATGG	
		BIP	AGTGTGCTTGGAGTGGCTTA-CAATCCCAGTTTGTAATATCGC	
		FIP	CACAGTACCACCAAACCAAAAAATA-TTGGTTAGCTATAGCTGTGA	
*Escherichia coli* O157	*wzy*	LB	TCCTTTTCTCTCCGTATTGAT	[8]
		LF	ATAATGATATATGAATAGAATGCGC	
		B3	ATAACTGATATTTTCATTTCGTGAT	
		F3	TCCCTTTAGGGATATATATACCTT	
		BIP	TTCCCAGCCACTAAGTATTGCAATATTTTTGAAAAAAACCCATAGCTCGA	
		FIP	TGCATCGGCCTTCTTTTTTGGTTTTAACGTATCATGCAATAAGATCA	
*Streptococcus agalactiae*	*sob*A	LB	AGGCGCTCTTAGCTGATGT	[9]
		LF	TGCATGGTGCTTATCATGATGT	
		B3	ACCACCGTTATTGATGACTG	
		F3	ATATGATGCGCTTGAGCC	
		BIP	ACATCCTGAAATTGGAGAAGACTTTTTTCCTGACGAATATCTTCTGGAAT	
		FIP	GAGCAGCATTTGCATTAGCAACATATTTTGATGCTGAGACAATGACAC	

### 3.2. Determination of Non-Specific Amplification

After the LAMP primers were synthesized by Sangon Biotech Co., Ltd (Shanghai, China), the non-specific amplification of the 12 in-house real-time LAMP assays as well as the commercial Isothermal Master Mix kit were determined via the corresponding negative controls with no genomic DNA. The experiment was performed before DNA extraction, therefore, cross pollution caused by DNA template as well as amplified products of LAMP reactions can be avoided, and the cause of false-positive results can be objectively judged.

The reaction mixtures and reaction conditions of the 12 in-house real-time LAMP assays were as described [4,5,6,7,8,9]. The 25 µL in house reaction system contained 1.6 µmol/L of each of the inner primers FIP and BIP, 0.2 µmol/L of each of the outer primers F3 and B3, and 0.8 µmol/L of each of the loop primers LF and LB. 1.2 mmol/L each dNTP, 6 mmol/L MgSO_4_, 1 × Bst DNA polymerase buffer (New England Biolabs, Beverly, MA, USA) (20 mmol/L Tris-HCl (pH = 8.8), 10 mmol/L KCl, 10 mmol/L (NH_4_)_2_SO_4_, 2 mmol/L MgSO_4_, 0.1% TritonX-100), 8 Units of Bst DNA large fragments (New England Biolabs). Depending on different experiments, 0 M, 0.6 M or 1.0 M of betaine (Sigma, St. Louis, MO, USA) were included, respectively. The LAMP assays were modified by adding 1 × EvaGreen and 1 × Rox, the experiment only on negative controls was carried out on StepOne™ System (Applied Biosystems, Foster City, CA, USA) at the described temperature (65 °C for the *inv*A of *Salmonella*, *rfbE* of *Escherichia coli O157*, *wzy* of *Escherichia coli* O26, *wzy* of *Escherichia coli* O45, *wzx* of *Escherichia coli* O103, *wzy* of *Escherichia coli* O111, *wzy* of *Escherichia coli* O121, *wzx* of *Escherichia coli* O145, *wzy* of *Escherichia coli* O157; 64 °C for *nuc* of *Staphylococcus aureus*; 63 °C for *sob*A of *Streptococcus agalactiae*) for 50 min, and each experiment was repeated three times.

The Real-time LAMP with Isothermal Master Mix was also carried out on StepOne™ System (Applied Biosystems), using 0.1 µM F3 and B3, 0.8 µM FIP and BIP, 0.4 µM LF, and LB, with 1 × Isothermal MasterMix containing a fluorescent intercalating dye [13]. The reactions of negative controls were held at 65 °C for 50 min with real-time fluorescence monitoring, and each experiment was repeated three times.

### 3.3. Bacteria Strains and DNA Extraction

Tweleve strains used for this study (Table 2) were obtained from the Food and Bioengineering College of Xuchang University. *Listeria monocytogenes* ATCC19115 was cultured overnight at 37 °C in Difco**™** Buffered *Listeria* Enrichment Broth Base (Becton, Dickinson and Company, San Jose, CA, USA) while the others in Luria-Bertani (LB) broth. Genomic DNA from these bacterial cultures was extracted using DNeasy^®^ Blood & Tissue Kit (Qiagen Inc., Valencia, CA, USA) according to the manufacturer’s instructions. The genomic DNA was used to determine the detection limits of 12 in-house real-time LAMP assays as well as the commercial kit Isothermal Master Mix.

### 3.4. Detection Limit Comparison

The above described reaction mixtures of 12 in-house real-time LAMP assays were combined with serial dilutions of *L. monocytogenes*, *Salmonella*, *Staphylococcus aureus*, *E. coli* O157, *E. coli* O26, *E. coli* O45, *E. coli* O103, *E. coli* O111, *E. coli* O121 and *Streptococcus agalactiae* DNA template ranging from 0.1–100 pg, respectively, the reactions were carried out on StepOne™ System (Applied Biosystems) at described temperature for 50 min, and each experiment was repeated four times, and the detection limits of 12 in-house real-time LAMP assays were determined.

For comparison, the detection limits of the commercial kit Isothermal Master Mix were determined by carrying out reactions according to the manufacturers’ instructions using a set of serially diluted DNA template of *L. monocytogenes*, *Salmonella*, *Staphylococcus aureus*, *E. coli* O157, *E. coli* O26, *E. coli* O45, *E. coli* O103, *E. coli* O111, *E. coli* O121 and *Streptococcus agalactiae* DNA template ranging from 0.1–100 pg, respectively, and each reaction was repeated four times.

## 4. Conclusions

Twelve reported in-house LAMP assays for the detection of pathogenic bacteria [4,5,6,7,8,9] have been compared with the commercialized Isothermal Master Mix kit in this study. False-positive results have been observed among all 12 in-house real-time LAMP assays, and it can be concluded from our experiments that the non-specific amplification is caused by primer dimers. It is difficult to avoid primer dimers and non-specific amplification when multiple sets of primers are used in the in-house LAMP assays. This is especially true when the concentrations of primers, Mg^2+^, dNTPs and DNA polymerase in reaction mixtures are as high as those used in real-time PCR [14]. The concentrations of these four factors must be strictly controlled to avoid non-specific amplification in real-time PCR as well as LAMP reactions [15].

False-positive results have not been found in real-time LAMP assays with the commercialized Isothermal Master Mix kit. It is speculated that the Isothermal Master Mix kit may contain some enhancing agents, which can decrease the non-specific amplification. The detection limits for most tested pathogenic bacteria are 1 pg DNA template, and the sensitivity of the commercial assay for *E. coli* O145 is 100 pg DNA template. In summary, the 12 in-house real-time LAMP assays were impractical for detection of the corresponding pathogenic bacteria, while the commercial Isothermal Master Mix kit was useful for detection of the most pathogenic bacteria.
